# Calcium Phosphates–Chitosan Composite Layers Obtained by Combining Radio-Frequency Magnetron Sputtering and Matrix-Assisted Pulsed Laser Evaporation Techniques

**DOI:** 10.3390/polym14235241

**Published:** 2022-12-01

**Authors:** Maria Elena Zarif, Sasa Alexandra Yehia-Alexe, Bogdan Bita, Irina Negut, Claudiu Locovei, Andreea Groza

**Affiliations:** 1 National Institute for Lasers, Plasma and Radiation Physics, 077125 Măgurele, Romania; 2 Faculty of Chemical Engineering and Biotechnologies, University Politehnica of Bucharest, 011061 Bucharest, Romania; 3 Faculty of Physics, University of Bucharest, 077125 Măgurele, Romania; 4 National Institute of Materials Physics, 077125 Măgurele, Romania

**Keywords:** calcium phosphate, chitosan, radio-frequency magnetron sputtering technique, matrix-assisted pulsed laser evaporation technique

## Abstract

In this work, we report the synthesis of calcium phosphate–chitosan composite layers. Calcium phosphate layers were deposited on titanium substrates by radio-frequency magnetron sputtering technique by varying the substrate temperature from room temperature (25 °C) up to 100 and 300 °C. Further, chitosan was deposited by matrix-assisted pulsed laser evaporation technique on the calcium phosphate layers. The temperature at the substrate during the deposition process of calcium phosphate layers plays an important role in the embedding of chitosan, as scanning electron microscopy analysis showed. The degree of chitosan incorporation into the calcium phosphate layers significantly influence the physico-chemical properties and the adherence strength of the resulted layers to the substrates. For example, the decreases of Ca/P ratio at the addition of chitosan suggests that a calcium deficient hydroxyapatite structure is formed when the CaP layers are generated on Ti substrates kept at room temperature during the deposition process. The Fourier transform infrared spectroscopy analysis of the samples suggest that the PO_4_^3−^/CO_3_^2−^ substitution is possible. The X-ray diffraction spectra indicated that the crystalline structure of the calcium phosphate layers obtained at the 300 °C substrate temperature is disturbed by the addition of chitosan. The adherence strength of the composite layers to the titanium substrates is diminished after the chitosan deposition. However, no complete exfoliation of the layers was observed.

## 1. Introduction

Calcium phosphates (CaPs) are constituents of teeth and bones and can be used for the coating of metallic implants. These materials are known for their biocompatibility and osteoconductive properties [1,2]. The CaP compounds frequently used for biological and medical purposes are: hydroxyapatite (Hap-Ca_10_(PO_4_)_6_(OH)_2_, β-tricalcium phosphate (β-TCP-Ca_10_(PO_4_)_2_), α-tricalcium phosphate (α-TCP-Ca_10_(PO_4_)_2_), or hydroxyapatite with calcium deficiency (Ca_10−x_(HPO_4_)_x_(PO_4_)_6−x_(OH)_2−x_, 0≤ x ≤ 1) [3]. Lately, these compounds have attracted attention due to their applications for dental or orthopedical implants [4].

Among the naturally derived biomaterials, chitosan was intensively studied as a coating material for improving bone formation [5]. It is a non-toxic, hemostatic, mucoadhesive, antioxidant, and biocompatible cationic polysaccharide polymer, derived from deacetylated chitin and made from glucosamine and *N*-acetyl glucosamine [5,6,7]. It is used as an antimicrobial agent for a broad spectrum of Gram positive and Gram negative bacteria [8]. Phosphorylated chitosan, one of the chitosan derivatives, is known to facilitate the dentin remineralization. The phosphate groups can bind calcium ions, forming nucleation sites, which can induce the biomimetic deposition of a CaP layer under stimulated physiological conditions [9].

Titanium (Ti) is commonly used in dentistry [10] and orthopedic applications [11] due to its biocompatibility and it remains the gold standard for implants. Despite this, implant failure was reported. Therefore, several implant coatings, which improve the osteointegration and the bacterial resistance, were developed over the years [5].

In the last years, a large number of techniques, such as magnetron sputtering, pulsed laser deposition, electrospinning, or thermal plasma spraying, were used for CaP deposition on various materials [12].

Magnetron sputtering is a physical deposition technique efficient to deposit various materials on different metallic or polymeric substrates [12]. This technique is able to deposit thin films on complex shapes [12,13], from different materials, relevant for biomedical applications [14]. In ref. [15,16], it is mentioned that uniform and dense CaP-based layers were successfully obtained by magnetron sputtering technique.

In a basic physical deposition, materials are sputtered from a target by energetic ions bombardment. This method was upgraded to the magnetron sputtering variant due to the fact that the basic sputtering process has low deposition rate. The magnetron is able to strongly confine the plasma in the target region, obtaining in this way a dense plasma. This aspect improves the ion bombardment of the target which in turn strongly increases the deposition rate and the properties of the deposited material [17]. An important aspect is that during the deposition process, substrate heating occurs. This phenomenon can be attributed, according to ref. [12], to the atom kinetic energy of the deposited material on substrate, to the energy released during mechanism of film growth, or to the gas atoms energy used for target sputtering.

The ability to fabricate a variety of biomaterials in the form of thin films, with reasonably uniform material spreading over relatively large regions, controlled film thickness, and good substrate adhesion is one of the many benefits of laser-based technologies [18,19]. Matrix-assisted pulsed laser evaporation (MAPLE) is a deposition laser-based technique which involves low material consumption and preserves the stoichiometry of the growing layers [8,20]. From the laser deposition methods, MAPLE provides a mild transfer of small and large molecular weight species from the condensed phase into the vapor one. Specific to this technique, the organic material is dissolved in a volatile solvent before being frozen at N_2_ temperature. The photochemical degradation of the organic substance can be decelerated or even stopped by hitting the frozen target with a pulsed laser beam whose energy is absorbed by the solvent and turned into thermal energy [21,22,23].

In this paper, we report the deposition and the physico-chemical characterization of CaP layers on Ti substrates, using different substrate temperatures by radio-frequency magnetron sputtering (RF-MS) technique and the deposition of chitosan layers on top of the CaP layers by MAPLE. The elemental composition and the molecular structure of CaP and CaP–chitosan (CaP_CS) layers were investigated by energy dispersive X-ray spectroscopy (EDX) and Fourier transform infrared spectroscopy (FTIR). The morphology of the CaP and CaP_CS layers was investigated by scanning electron microscopy (SEM). The surface chemistry of the coatings was investigated by X-ray photoelectron spectroscopy (XPS). The structural characterization was conducted by X-ray diffraction. The adherence strength of the CaP and of the CaP_CS layers was evaluated by tape adhesion test.

## 2. Materials and Methods

### 2.1. Materials

#### 2.1.1. Titanium Substrates

The Ti samples were cut in pieces of (1 mm × 1 mm) from an annealed titanium foil, with a thickness of 0.5 mm, purchased from Alfa Aesar. Mirror-like surfaces were obtained by optical polishing.

#### 2.1.2. Calcium Phosphate Tribasic Powder

The calcium phosphate tribasic (CaP) powder (CAS number: 12167-74-7), with the chemical formula Ca_10_(OH)_2_(PO4)_6_, a molecular weight of 1004.67 g/mol, and a Ca/P ratio of 1.67, was purchased from Alfa Aesar. Sputtering targets with diameters of 5 cm and approximately 4 mm thickness were prepared from the CaP powder by mechanical pressing.

#### 2.1.3. Chitosan

Low molecular weight chitosan (CAS number: 9012-76-4), with the chemical formula C_12_H_24_N_2_O_9_, a molecular weight ranged between 50,000–190,000 Da, prepared by the deacetylation of chitin obtained from shrimp shells (deacetylation degree of 75–85%) was purchased from Sigma-Aldrich.

### 2.2. Deposition Techniques

#### 2.2.1. Calcium Phosphate Deposition by Radio-Frequency Magnetron Sputtering

**Figure 1:**
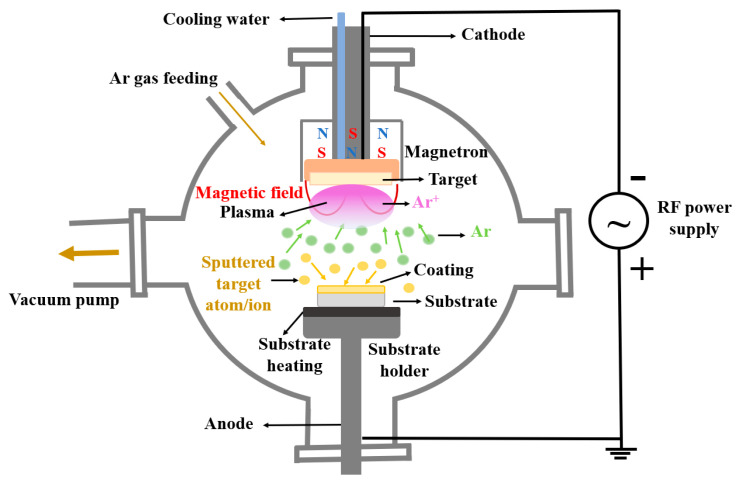
Schematic representation of RF-MS experimental setup.

The deposition of the CaP layers was conducted in Ar gas flow using the RF-MS technique using a 2 inch magnetron plasma source purchased from K.J. Lesker Company coupled with an RF supply power generator (13.56 MHz). During the deposition process, the RF supply power was set to 100 W and the Ar gas flow to 5 mL_n_/min. The base pressure of the vacuum chamber was ~10^−5^ mbar. The working pressure was ~10^−2^ mbar. The distance between the magnetron head and the substrate holder was about 8 cm. The substrate holder was grounded and heated during the deposition process by a homemade electronically controlled oven from room temperature (25 °C) (CaP_1) up to 100 (CaP_2) and 300 °C (CaP_3). The deposition time was set to 5 h. The schematic representation of the RF-MS experimental setup is presented in Figure 1.

The CaP deposition rate was measured using a quartz crystal microbalance, purchased from INFICON Holding AG Company, placed centrally to the plasma source before the CaP layer generation. The deposition rate was about 0.1 Å/s and the thickness of the deposited layers at room temperature was about 160 nm.

#### 2.2.2. Chitosan Deposition by Matrix-Assisted Pulsed Laser Evaporation

**Figure 2:**
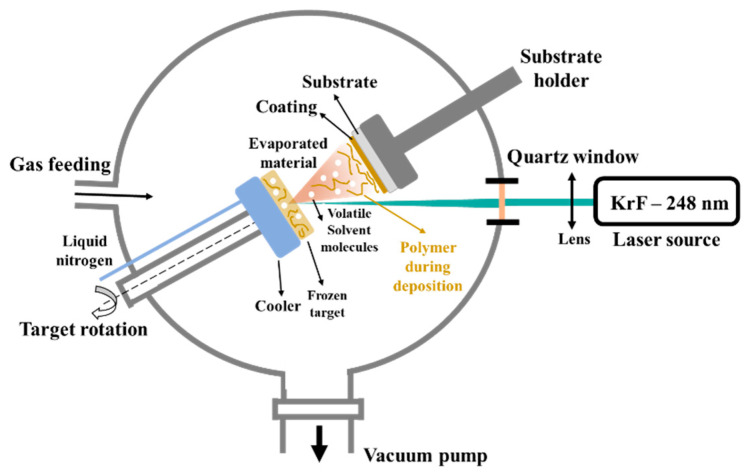
Schematic representation of MAPLE experimental setup.

The MAPLE solid targets were prepared by freezing at N_2_ temperature a 2% chitosan solution. The solution was made by mixing chitosan with distilled water, using a magnetic stirring for homogenization, adding in the meantime 200 µL of acetic acid. Due to the low molecular weight, non-dissolved chitosan parts were not observed and no filtering was required. The radiation from a KrF* (λ = 248 nm, τFWHM = 25 ns) (model COMPexPro 205 Lambda Physics-Coherent) excimer laser source swept the surface of the frozen target at a laser fluence of 300 mJ/cm^2^ and at a repetition rate of 10 Hz. During the deposition, the target was rotated with 50 rpm Hz to avoid drilling and to ensure a uniform coating. All depositions were conducted at room temperature in a pressurized deposition chamber (0.1 Pa background pressure) and at a 5 cm target-to-substrate separation distance. The thin films were obtained by applying 100,000 subsequent laser pulses. During the deposition, the target was maintained at a low temperature by means of a cooling system, continuously supplied with N_2_. The layers were deposited onto CaP-coated Ti substrates. The schematic representation of the MAPLE experimental setup is presented in Figure 2.

### 2.3. Characterization Techniques

The surface chemistry was analyzed by X-ray photoelectron spectroscopy (XPS) using a K-Alpha Thermo Scientific (ESCALAB™ XI) spectrometer. A monochromatic Al Kα X-ray source (1486.68 eV) and an X-ray spot of 900 μm diameter were used to acquire the XPS spectra. The charging effect compensation was ensured by an electron flood gun. For the correction of charging effects during acquisition, all spectra were calibrated using the C 1s peak at 284.6 eV. Energy step sizes of 1 and 0.1 eV were used for the survey and high-resolution spectra, respectively. The recording and the processing of the survey and high resolution XPS spectra were performed using advanced Avantage software (Thermo Avantage v5.976, Thermo Fisher Scientific, East Grinstead, UK). The XPS peak fitting analyses were performed using the MagicPlotPro software and a nonlinear least squares algorithm. We used a Gaussian-type profile curve.

The identification of functional groups was ensured by Fourier transform infrared spectroscopy (FTIR) analysis using a Perkin-Elmer SP 100 FTIR spectrometer equipped with an attenuated total reflection unit. The spectra were acquired in the range of 4000–500 cm^−1^ with a resolution of 4 cm^−1^. The curve-fitting for the FTIR spectra was performed as mentioned in ref. [24].

X-ray diffraction (XRD) was employed for the structural characterization of samples, as well as for the CaP powder and magnetron sputtering target after layers deposition. The XRD data were acquired from a Bruker D8 Advance diffractometer working with CuKα radiation (λ_Ka_ = 1.54 Å) at ambient conditions. To minimize the signal from Ti substrate and to increase the X-ray photons path into prepared layers all the samples were analyzed in the grazing incidence geometry (GIXRD) in the interval 2θ = 20–55° with an angular step of 0.02°. Moreover, the initial CaP powder and the homemade magnetron sputtering target were characterized at XRD in symmetric geometry in a larger interval of 2θ = 20–100°.

The surface morphology of the samples was evaluated by scanning electron microscopy (SEM) and the elemental composition by energy dispersive X-ray spectroscopy (EDX). For the SEM analysis, the samples were coated with a thin layer (5 nm) of Au using a magnetron sputtering device equipped with an Au solid target purchased from Cressington Scientific Instruments. The electrical discharge was performed with argon gas and DC current generator. The desired thickness was controlled by a quartz crystal microbalance incorporated in the sputter coater device with a resolution of 0.1 nm and measurement range up to 1000 nm. The cross-section SEM images were performed by positioning the samples at a 3-degree inclination angle to the vertical position. The surfaces of the CaP and CaP_CS layers deposited on Ti substrates were analyzed using a ThermoFisher Apreo S scanning electron microscope working in high vacuum modes, at 1.3 × 10^−3^ Pa and 10 kV, and EDX detector (SiLi). The energy dispersive X-ray spectroscopy (EDX) investigation of the layers was acquired at high voltages applied on the field emission gun (FEG), at 10 kV. Surface texture modifications of CaP coatings after the adding of chitosan were visualized by the 3D surface plot analyses performed by using the Image J software (ImageJ 1.51j8, National Institutes of Health, Bethesda, MD, USA).

The adherence of the CaP and CaP_CS layers was investigated following the ASTM standard procedure [25] characteristic to adhesion tape tests (D3330 test method for peel adhesion of pressure-sensitive tape). We used a 3M Performance Flatback Tape 2525 tape with a peel adhesion of 7.5 N/cm and tensile strength of 85.8 N/cm.

## 3. Results and Discussion

### 3.1. X-ray Photoelectron Spectroscopy

The surface chemistry was evaluated by XPS for the CaP and CaP_CS coatings.

#### 3.1.1. XPS Analysis of CaP Coatings as a Function of Substrate Temperature

**Figure 3:**
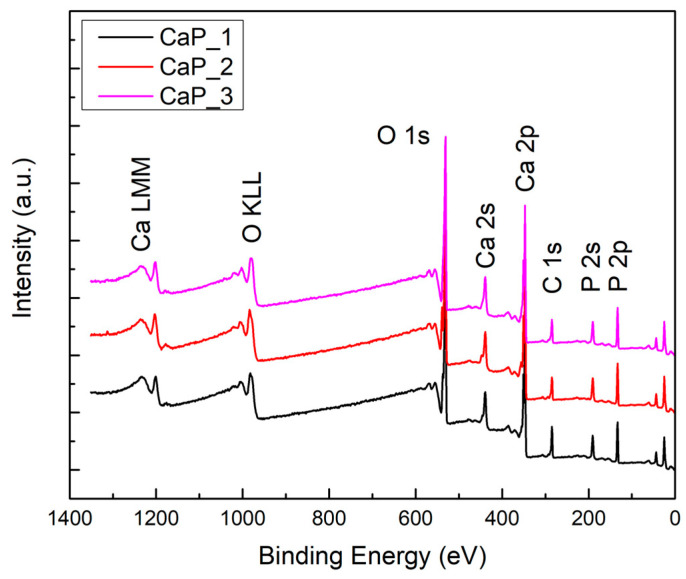
XPS survey spectra of CaP coatings.

The XPS survey spectra confirmed the presence of Ca, P, and O—chemical elements characteristic for CaPs, and Ti—from the substrate (see Figure 3).

The high-resolution XPS spectra (Ca 2p, P 2p, O 1s, C 1s, and Ti 2p) acquisition was required in order to evaluate the chemical structure of the CaP coatings. All the high-resolution spectra were fitted and the binding energies of the deconvoluted peaks are presented in Table 1.

**Figure 4:**
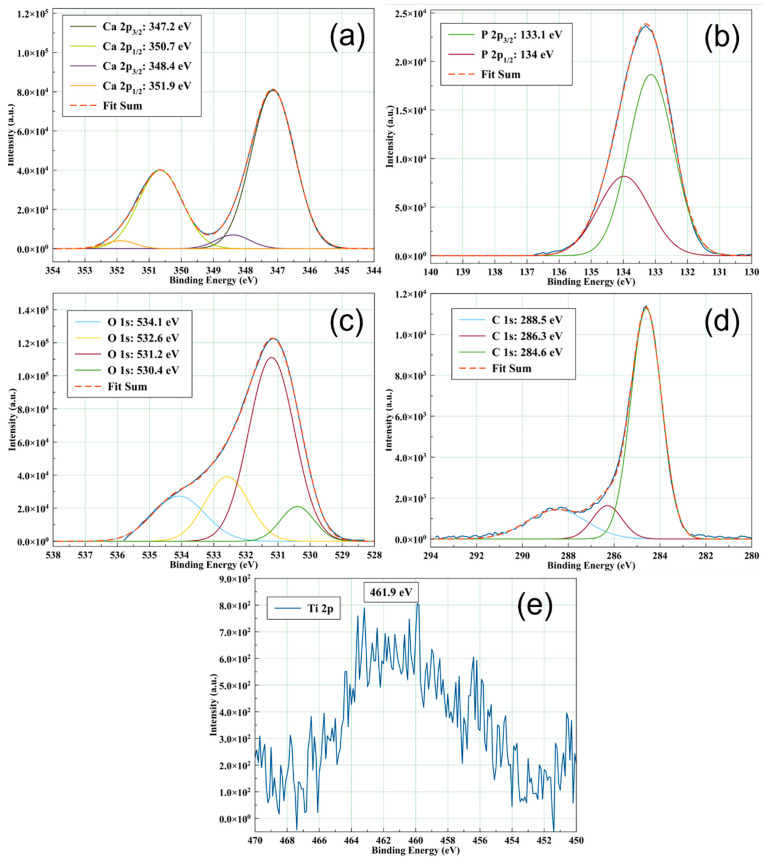
XPS high-resolution deconvoluted spectra of CaP_1 sample: (**a**) Ca 2p, (**b**) P 2p, (**c**) O 1s, and (**d**) C 1s and XPS high-resolution spectrum of (**e**) Ti 2p.

The deconvoluted spectra of Ca 2p, P 2p, O 1s, and C 1s for the CaP coating deposited by RF-MS technique without substrate heating are presented in Figure 4. Peaks corresponding to Ca 2p_3/2_ (347.2 and 348.4 eV), P 2p_3/2_ (133.1 eV), O 1s (530.4, 531.2, 532.6, and 543.1 eV), and C 1s (284.6, 286.3, and 288.5 eV) were identified in the XPS deconvoluted spectra of the CaP_1 coating. These peaks were assigned to Ca-Ca, Ca-OH, and Ca-O in Hap (347.2 eV) and Ca-O in CaO (348.4 eV) [26] for Ca 2p_3/2_ and P-O in PO_4_ of Hap (133.1 eV) for P 2p_3/2_ [26]. The deconvoluted peaks of Ca 2p and P 2p maintain the 1:2 area ratios of Ca 2p_1/2_: Ca 2p_3/2_, and P 2p_1/2_: P 2p_3/2_, namely [27] and Δ (Ca 2p_3/2_ − Ca 2p_1/2_) = 3.5 eV and Δ (P 2p_3/2_ – P 2p_1/2_) = 0.84 eV [26]. The values for Ca 2p_1/2_ and P 2p_1/2_ are presented in Table 1.

The O 1s peaks were assigned to O-Ca in HAp (530.4 eV) [26], O-P, O-H, and O-C in PO_4_^3−^, OH^-^ and CO_3_^2−^ (531.2 eV) [26], HPO_4_^2−^ (532.6 eV) [28], and P=O (534.1 eV) [29] in the XPS deconvoluted spectra of the CaP coating deposited without heating the substrate. Boyd et al. reported the presence of HPO_4_^2−^ at 532.2 or 533 eV. However, they corrected the sample charging effects using a binding energy of 285 eV for the lowest binding energy component of C 1s, while we set this value at 284.6 eV [28]. Therefore, we may assign the peak at 532.6 eV to HPO_4_^2−^. Uskoković reported that the binding energy ranged between 534.01 and 534.12 eV can be assigned to P=O. In this case, the energy calibration (C 1s) for all spectra was performed at 284.5 eV [29]. Considering this, we may assign the peak at 534.1 eV to P=O. The C 1s peaks were assigned to C-C adventitious carbon/organic species (284.6 eV) [26,29], C-O adventitious carbon (286.3 eV) [29], and C-O in CO_3_^2−^ (288.5 eV) [26,29]. The Ti 2p XPS line appears as a broad peak at 461.9 eV probably due to titanium oxide formation at layer/substrate interface during the deposition process and its diffusion into the layer volume up to the surface.

The XPS binding energies corresponding to the CaP coatings deposited at a substrate temperature of 100 and 300 °C are presented in Table 1. Slight shifts (~0.2 eV) of Ca 2p, P 2p, O 1s, and C 1s XPS peaks can be observed. Therefore, we suppose that the high substrate temperature during the deposition process influences the chemical structure of the layers surface. Moreover, the intensity of Ca 2p_3/2_ and P 2p_3/2_ XPS lines attributed to Hap structure slightly increases as the substrate temperature increases during the deposition process. This can be an indication that a high substrate temperature can favor the formation of Hap structure.

#### 3.1.2. XPS Analysis of CaP_CS Coatings

**Figure 5:**
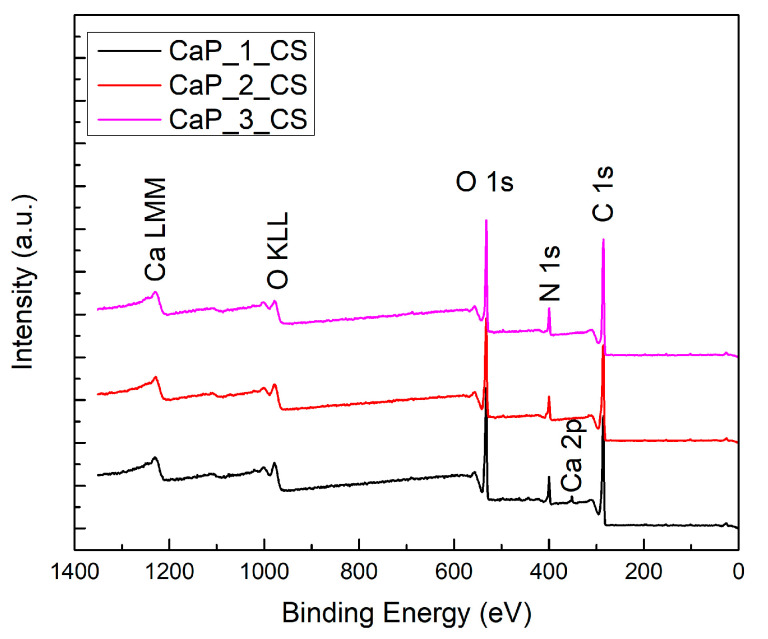
XPS survey spectra of CaP_CS coatings.

The results obtained after the XPS spectral analysis of CaP_1_CS, CaP_2_CS, and CaP_3_CS samples are presented in the following. The XPS full spectra (see Figure 5) of the analyzed samples indicate the presence of the elements specific to chitosan: C, N, and O and for CaP_1_CS sample the Ca 2p peak is visible.

The high-resolution spectra were fitted and the binding energies of the deconvoluted peaks are presented in Table 2.

**Figure 6:**
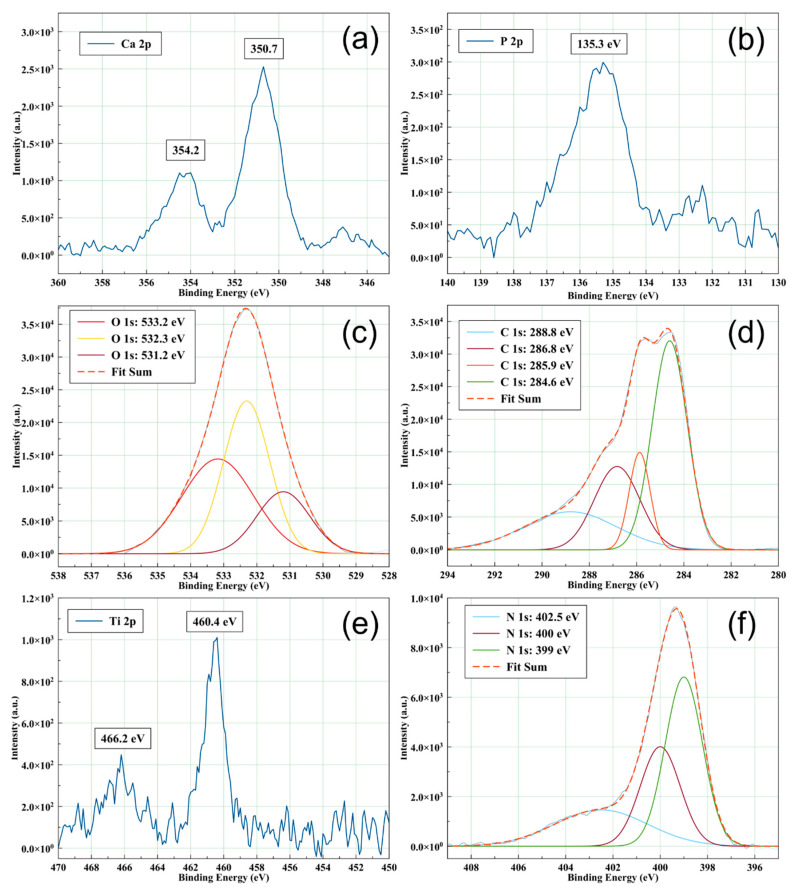
XPS high-resolution spectra: (**a**) Ca 2p, (**b**) P 2p, and (**e**) Ti 2p and XPS high-resolution deconvoluted spectra: (**c**) O 1s, (**d**) C 1s, and (**f**) N 1s for the CaP_1_CS.

The high resolution XPS spectra of CaP_1_CS sample present the following lines: Ca 2p, P 2p, C 1s, O 1s, N 1s, and Ti 2p. The identification of Ca 2p (see Figure 6a) and P 2p (see Figure 6b) XPS lines indicate that the chitosan is embedded in the structure of CaP layer. The Ca 2p_3/2_ and Ca 2p_1/2_ lines are shifted to higher energies up to 350.7 eV and 354.2 eV, respectively. These shifts of the Ca 2p XPS lines indicate the oxidation of the surface [30]. The P 2p peak is centered at 135.3 eV (see Figure 6b) suggesting also the oxidation of the surface [31]. Moreover, the Ti 2p peak is also evidenced. The Ti 2p peaks which appear at 460.4 eV and 466.2 eV (see Figure 6e) were previously attributed to TiO_2_ structure [32].

The deconvoluted XPS O1s peak for CaP_1_CS (see Figure 6c) is formed by three bands: 531.2 eV (O-P in PO_4_^3−^), 532.3 eV (polysaccharide backbone of chitosan) and 533.2 eV (O-H from chitosan) [33]. We suppose that the 533.2 eV peak can be due to adsorption on the surface of the CaP_1_CS layer of H_2_O molecules [30] resulted during the evaporation of chitosan in the MAPLE deposition process.

The C 1s peak observed in the spectrum of CaP_1_CS contain 4 subpeaks (see Figure 6d) positioned at 284.6 eV (C-C/C-H), 285.7 (C-O/C-OH), 286.8 eV (C-O/C-N/O-C-O) [34], and 288.8 eV (-C=O) [35]. Moreover, the intensity of the deconvoluted band from 288.8 eV (see Figure 6d) is higher than the intensity of 288.5 eV attributed to C-O in CO_3_^2−^ (see Figure 4d), suggesting the increase of carbon oxides content at the surface of CaP_1_CS layer, possible due to CO_3_^2−^ formation.

The N1s XPS high resolution spectrum (see Figure 6f) contains 3 subpeaks located at 399, 400, and 402.5 eV. The first peak is attributed to N-C bonds, the second one to C-NH_3_^+^ bending (protonated NH_3_^+^). The peak from 402.5 eV can suggest the interaction of NH_3_^+^ with CaP as previous was reported for chitosan-montmorillonite composites [34].

The absence of substrate heating during the deposition of CaP_1 layer in the RF magnetron discharge conduce to the presence of Ca 2p and P 2p XPS lines at the surface of the CaP_1_CS sample. High resolution XPS analysis of CaP_2_CS and CaP_3_CS does not show the presence of Ca 2p and P 2p lines. A possible explanation could be given if we consider that the chitosan is deposited mainly on the surface of CaP layers, without being totally embedded in it.

### 3.2. Fourier Transform Infrared Spectroscopy

The FTIR was required in order to identify the absorption bands characteristic for the CaPs chemical groups, namely PO_4_^3−^, OH^−^, and CO_3_^2^.

For HAp, the main chemical groups, which can be confirmed by FTIR, are PO_4_^3−^, OH^−^, and CO_3_^2−^. However, for non-stoichiometric HAp, HPO_4_^2−^ may also appear [3]. In general, for the tetrahedral phosphate ion (PO_4_^3−^), four active vibrations are expected: ν_1_ (symmetric stretching mode): 963–961 cm^−1^, ν_2_ (bending mode): 472 cm^−1^, ν_3_ (asymmetric stretching mode): 1090–1040 cm^−1^_,_ and ν_4_ (bending mode): double band 602–601 and 575–560 cm^−1^. The presence of the hydroxyl group (OH^−^) is also evidenced by several absorption bands at 3570 cm^−1^ (stretching mode of lattice water), ~1640 cm^−1^ (bending mode of adsorbed water), and 631 cm^−1^ (ν_L_-libration mode). The presence of CO_3_^2−^ is highlighted by absorption bands around 875 cm^−1^ and in the 1600–1400 cm^−1^ wavenumber range. Depending on their positions, A-type (CO_3_^2−^ substitutes OH^-^), B-type (CO_3_^2−^ substitutes PO_4_^3−^), or AB-type carbonation can be evidenced [3,36,37,38].

**Figure 7:**
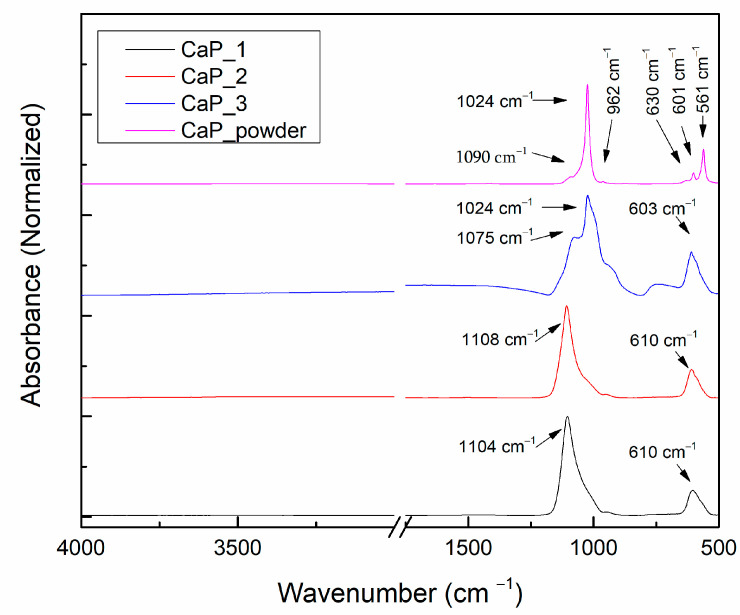
FTIR spectra of the CaP powder and of the CaP coatings deposited at different substrate temperatures (CaP_1, CaP_2, and CaP_3).

Figure 7 presents the FTIR spectra of the CaP powder and of the CaP coatings deposited at different substrate temperatures (ambient temperature, 100 °C, and 300 °C). As it can be observed in Figure 7, no absorption bands were identified in the 3600–3100 cm^−1^ wavenumber range or around 1640 cm^−1^, which indicate no OH groups. Moreover, absorption bands characteristic for CO_3_^2−^ were not observed, which indicates that carbonation does not occur during the magnetron sputtering deposition of the CaP coatings. The absorption band at 630 cm^−1^ can be assigned to the librationmode of apatite OH^−^ (ν_L_), while the absorption bands at 601 and 561 cm^−1^ can be assigned to the bending mode of apatite PO_4_ (ν_4_) in CaP powder (pink line in Figure 7) [36]. In the IR spectra of the analyzed samples (see Figure 7), all the vibration modes characteristics to PO_4_^3−^ can be observed and some wavenumber shifts that occur as function of the deposition conditions. For this reason and in order to identify all the changes that appear during the CaP films formation, we performed a peak fitting analysis.

#### 3.2.1. FTIR Analysis of CS Powder

**Figure 8:**
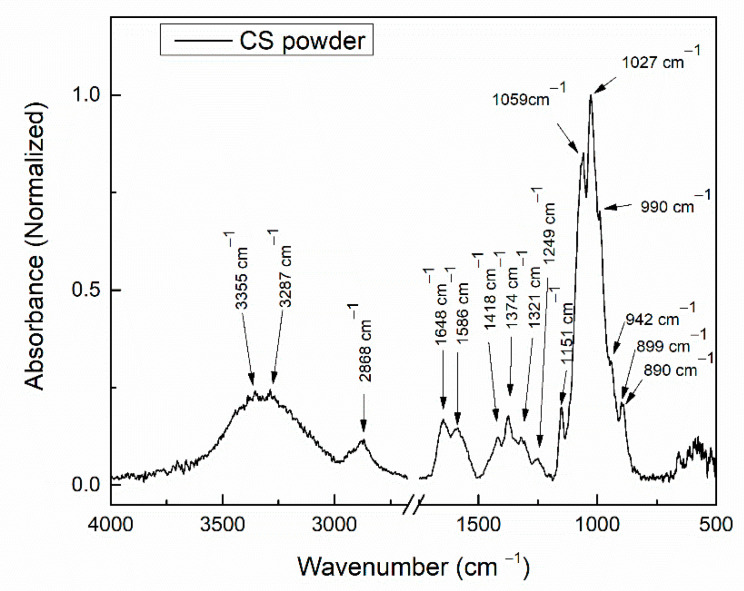
FTIR spectra of CS powder.

Figure 8 presents the FTIR spectra of the chitosan powder in the wavenumber range 4000–500 cm^−1^.

Absorption bands characteristic for chitosan were identified in the FTIR spectrum of the chitosan powder. A broad absorption band, with two maxima at 3355 and 3287 cm^−1^ could be assigned to the stretching vibrations of polymeric OH and to the stretching vibrations of N-H bonds [39]. The absorption band at 2868 cm^−1^ can be assigned to the asymmetric stretching of C-H [40,41]. The absorption band at 1648 cm^−1^ corresponds to the stretching of C=O of amide I [8,41], while the absorption band at 1586 cm^−1^ can be assigned to the bending of N-H [39]. The absorption bands at 1418, 1374, and 1321 cm^−1^ can be assigned to the bending vibration of C-H in -CH_2_ [41] and -CH_3_ [8,41], and C-N stretching of amide III [41], respectively.

The band at 1151 cm^−1^ corresponds to the asymmetric stretching of C-O-C bonds of the chitosan structure [8]. Absorption bands around 1059, 1027, and 990 cm^−1^ correspond to the symmetric stretching of C-O in C-OH, C-O-C, and CH_2_OH groups [8,41]. The bands at 1249 and 942 cm^−1^ can also be assigned to the stretching vibration of C-O [42]. The absorption band at 899 cm^−1^ corresponds to the wagging of C-H bonds (bending out of the plane of the monosaccharide ring) [8,41]. The absorption band at 890 cm^−1^ is characteristic to the glucopyranose ring [43].

#### 3.2.2. Peak Fitting Analysis of FTIR Spectrum of CaP Powder

**Figure 9:**
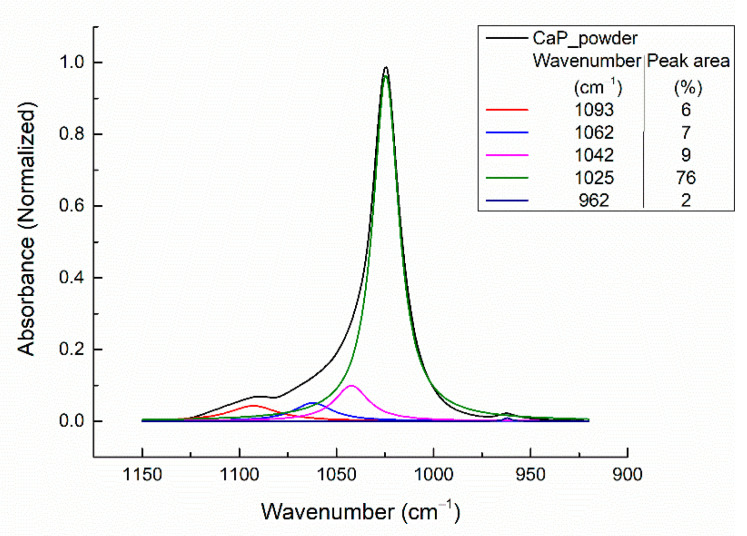
Deconvoluted IR bands of CaP sputtering target.

The deconvoluted IR bands of the CaP powder in the 1150–900 cm^−1^ spectral range and the peak area percentages are presented in Figure 9.

In the wavenumber range 1300–800 cm^−1^, five absorption bands were identified after peak fitting analysis. The absorption band at ~962 cm^−1^ can be assigned to the symmetric stretching mode of the apatite PO_4_^3−^ (ν_1_), while the bands in the range 1100–1000 cm^−1^, namely 1093, 1062, 1042, and 1025 cm^−1^ can be assigned to the asymmetric stretching mode of PO_4_^3−^ (ν_3_) [3].

Berzina-Cimdina and Borodajenko [3] presented several absorption bands values characteristic for CaPs. For the symmetric stretching mode of the apatite PO_4_^3−^ (ν_1_), the values ranged between 961 and 964 cm^−1^. The highest value (964 cm^−1^) was reported for the commercially available HAps. 

For the commercially available HAps, the reported wavenumber ranges for the absorption bands characteristic for PO_4_^3−^ were 1156–1000 cm^−1^[3]. However, the main bands characteristic for PO_4_^3−^ (ν_3_) in HAp are 1090 and 1040 cm^−1^ [3]. Our results indicate two IR deconvoluted bands at 1093 and 1042 cm^−1^. The strong absorption band from 1025 cm^−1^ [44] and the one from 1062 cm^−1^ [45] are also specific to the apatite structure (ν_3_ PO_4_^3−^).

#### 3.2.3. Peak Fitting Analysis of FTIR Spectra of CaP Coatings as Function of Substrate Temperature

**Figure 10:**
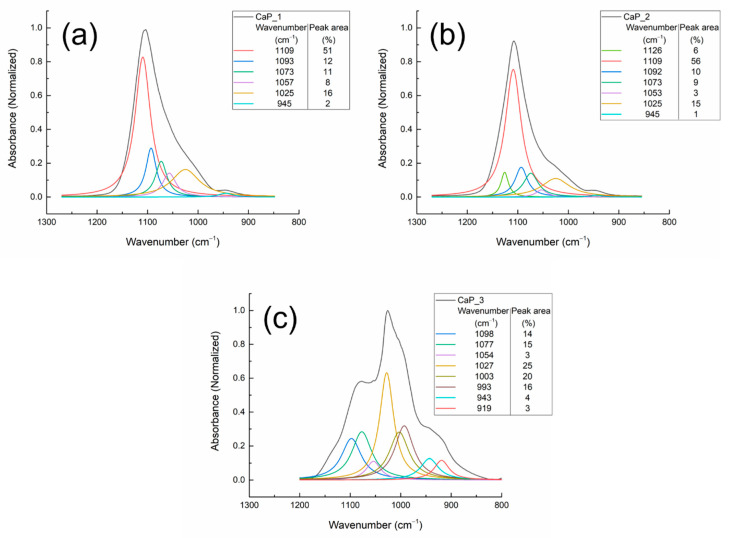
Deconvoluted FTIR spectra of the CaP coatings deposited at different substrate temperatures: (**a**) CaP_1, (**b**) CaP_2, and (**c**) CaP_3 in the 1300–800 cm^−1^ wavenumber range.

The deconvolution results of the CaP coatings FTIR bands deposited at 100 W and different substrate temperatures are presented in Figure 10 and Table 3.

For the wavenumber range 1300–800 cm^−1^, the absorption bands characteristic for HAp were identified for all samples.

The absorption bands at 1126 and 1109 cm^−1^ identified for the CaP coating deposited at ambient and 100 °C temperatures could be assigned to the asymmetric stretching mode (ν_3_) of PO_4_^3−^. As these bands are not present in the spectrum of CaP layer generated when the substrate was heated to 300 °C, we suppose that the appearance 1126 and 1109 cm^−1^ bands could be attributed to a major decomposition of HAp structure during the plasma deposition process and incorporation of HPO_4_ in HAp structure [46]. Moreover, in [47], it was mentioned that possible Ti-O-P interlinkings could be responsible of the findings of PO_4_^3-^ vibrations at these wavenumbers.

In the wavenumber range 1100–800 cm^−1^, five absorption bands, common for all samples (Table 3), were identified in the FTIR spectra. For the CaP coating deposited on Ti substrates without heating the substrate, these bands are: 1093 and 1073 cm^−1^ [38,44,48,49], 1057 [50] and 1025 cm^−1^ [44], assigned to asymmetric stretching mode (ν_3_) of PO_4_^3−^ in HAp, and 945 cm^−1^, symmetric stretching mode (ν_1_) of PO_4_^3−^ in HAp [45].

Depending on the substrate temperature during the RF-MS deposition, several shifts were observed in the reported values from Table 3. Moreover, following the peak area percentages, it results that as the temperature increases, chemical and physical modifications are observed in the layers.

#### 3.2.4. Peak Fitting Analysis of FTIR Spectra of CaP_CS Samples

**Figure 11:**
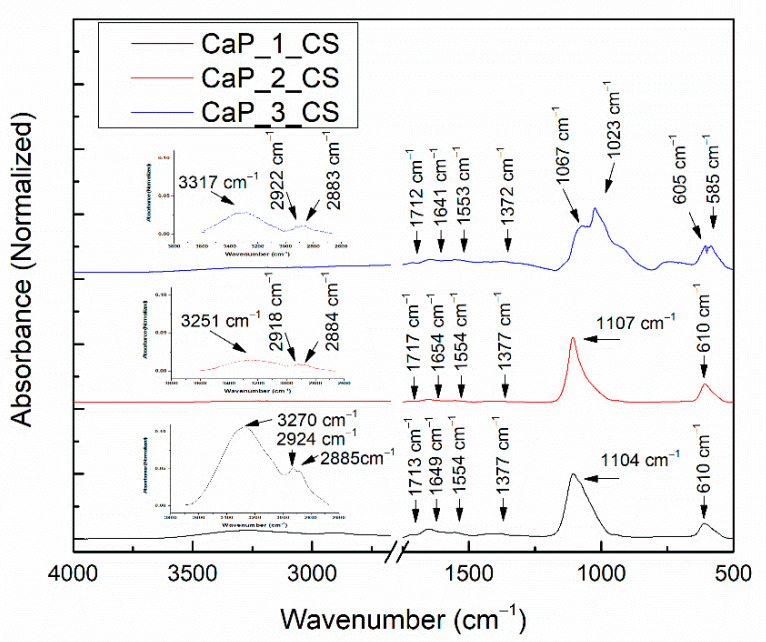
FTIR spectra of CaP_CS layers.

In Figure 11, the FTIR spectra of CaP_CS layers are presented. Table 4 indicates the IR bands specific to CS.

Absorption bands characteristic for chitosan were also identified in the FTIR spectra of the CaP_CS layers. The assignments of the absorption bands are presented in Table 4. When compared to the FTIR spectrum of the chitosan powder, two new absorption bands were identified in the FITR spectra of the CaP_CS layers at 2924 cm^−1^ (CaP_1_CS), characteristic for the symmetric stretching of C-H bond [8,41] and at 1713 cm^−1^ (CaP_1_CS), characteristic to the C=O [33].

Moreover, in the wavenumber range 1800–1200 cm^−1^, some differences in the IR band positions attributed to chitosan are observed. The bending vibration of N-H from 1586 cm^−1^, bending vibration of C-H in -CH_2_ from 1418 cm^−1^, and bending vibration of C-H in -CH_3_ from 1374 cm^−1^ (in the FTIR spectrum of CS powder) are shifted to 1554, 1426, and 1377 cm^−1^ in the FTIR spectrum of CaP_1_CS layer. These shifts can indicate that an interaction between chitosan and CaP layers takes place. Moreover, the band at 1426 cm^−1^ may also be assigned to the asymmetric stretching of CO_3_^2−^ [8]. This may indicate the substitution of PO_4_^3−^ by CO_3_^2−^ [51]. These results are in agreement with the XPS data, which indicated that at the surface of CaP_1_CS layer the content of carbon oxide increased (see Figure 4d and Figure 6d).

Depending on the substrate type (CaP coatings deposited on Ti at different substrate temperatures), several shifts were observed (see Table 4).

**Figure 12:**
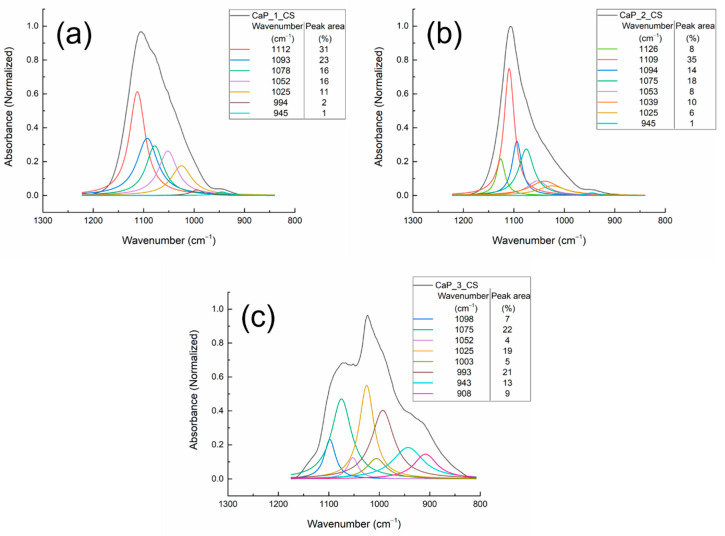
Deconvoluted FTIR spectra of the (**a**) CaP_1_CS, (**b**) CaP_2_CS, and (**c**) CaP_3_CS in the 1300–800 cm^−1^ wavenumber range.

The deconvolution results of the FTIR bands of the CaP_CS coatings obtained by combining the RF-MS technique and MAPLE technique are presented in Figure 12 and Table 3.

**Table 3:** The assignment of FTIR absorption bands for the CaP and CaP_CS coatings.

Wavenumber (cm^−1^)			Wavenumber (cm^−1^)			FTIR Band Assignment	Ref.
CaP_1	CaP_2	CaP_3	CaP_1_CS	CaP_2_CS	CaP_3_CS		
1109	1126 1109		1112	1126 1109		ν_3_ PO_4_^3−^ HPO_4_ incorporation	[46]
1093 1073 1057 1025	1093 1074 1053 1025	1098 1077 1054 1028 1003	1093 1078 1052 1025	1094 1075 1053 1025	1098 1075 1052 1025 1003	ν_3_ PO_4_^3−^ in Hap	[38,44,48,49,50,52]
				1039		P-O-C/P-O bond vibrations	[39]
		993 919	994		993 908	ν_3_ PO_4_^3−^/ν_3_-ν_1_ coupling/symmetric stretching of C-O	[53]/[8]
945	945	943	945	945	943	ν_1_ PO_4_^3−^ in Hap/stretching vibration of C-O	[45]/[42]

**Table 4:** The assignment of FTIR absorption bands for the CaP_CS coatings in the wavenumber domains: 4000–2500 cm^−1^ and 1800–1200 cm^−1^.

Wavenumber (cm^−1^)			FTIR Band Assignment	Ref.
CaP_1_CS	CaP_2_CS	CaP_3_CS		
3270	3251	3317	polymeric O-H stretch or N-H	[39]
2924	2918	2922	C-H symmetric stretching	[8,41]
2885	2884	2883	C-H asymmetric stretching	[40,41]
1713	1717	1712	C=O	[33]
1649	1654	1641	stretching of (-C=O-) of amide I group	[8,41]
1554	1554	1553	bending of N-H	[39]
1426	1428	1424	bending of C-H in (-CH_2_)/asymmetric stretching of CO_3_^2−^	[41]/[8]
1377	1377	1372	bending of C-H in (-CH_3_)	[8,41]
1321	1318	1319	C-N stretching of amide III	[41]

For the wavenumber range 1300–800 cm^−1^, absorption bands characteristic for Hap, were identified for all samples. However, as observed in the FTIR spectrum of the chitosan powder (Figure 8), absorption bands characteristic to chitosan also appear in this wavenumber range. Therefore, the bands characteristic for Hap and chitosan can overlap.

When compared to the values reported for the CaP coatings, several shifts were observed for the chitosan coated samples (see Table 3). In the sample CaP_1_CS, the presence of the 994 cm^−1^ IR band indicates the presence of chitosan. A new absorption band, at 1039 cm^−1^, was observed for the chitosan coating deposited on the CaP coating obtained by heating the substrate at 100 °C during the RF-MS deposition process. This band could be assigned either to P-O vibrations or to P-O-C bonds formations [39], which means that interaction between HAp and chitosan take place.

The absorption band at 1151 cm^−1^, identified in the FTIR spectrum of the chitosan powder (see Figure 8) was not observed in the deconvoluted FTIR spectra of the CaP_CS layers (see Figure 12), indicating the interaction between HAp and chitosan. Szatkowski et al. reported that the deformation or the disappearance of the ether bond in the pyranose ring at 1153 cm^−1^ is evidence of the chemical interaction between HAp and chitosan [54].

The modifications of peak area percentages can also indicate some structural changes in the structure of the composite layer.

The deconvolution band from 1057 cm^−1^ attributed to asymmetric stretching mode (ν_3_) of PO_4_^3−^ in HAp in the spectrum of CaP_1 sample (see Figure 10a) is shifted to 1052 cm^−1^ in the deconvoluted spectrum of CaP_1_CS (see Figure 12a). The peak area increases from 8% to 16%. The HWHM (half width at half maximum) increases from 15.9 cm^−1^ for CaP_1 to 22.4 cm^−1^ for CaP_1_CS, indicating therefore the broadening of the peak and the interaction between HAp and chitosan. This finding may suggest that chitosan was incorporated in the CaP_1 layer. In the case of CaP_2 (1053 cm^−1^) and CaP_3 (1054 cm^−1^) samples, the absorption bands shifts and the increase of the peak areas were not significant (see Figure 10b,c and Figure 12b,c). However, the HWHM increases from 13.1 cm^−1^ for CaP_2 to 24.8 cm^−1^ for CaP_2_CS, indicating the interaction between HAp and chitosan. For the CaP_3 the HWHM decreases from 17.2 cm^−1^ to 12.9 cm^−1^. Danilchenko et al. reported that the broadening of the 1050 cm-1 band indicates the interaction between chitosan and the phosphate groups [55].

The interactions between chitosan and HAp can be both physical (hydrogen bonds between chitosan and OH^−^, Ca^2+^, and PO_4_^3−^) [56] and chemical (coordination bonds between Ca (II) of HAp and -NH_2_, -OH, and oxygen of the linkage of chitosan) [57]. Moreover, O of PO_4_ can also be coordinated with N-H of chitosan [57]. Generally, the most reported interactions between HAp and chitosan are the hydrogen bonds formed between -OH of HAp and -OH and -NH_2_ of chitosan and the coordination bonds between Ca (II) of HAp and -NH_2_ of chitosan [55,58,59,60,61].

The FTIR results confirm that chitosan was successfully deposited by MAPLE on the CaPs coatings. Considering the fact that the chitosan deposition was conducted identically for all samples, the wavenumber shifts and peak areas changes identified in the deconvoluted spectra of CaP_CS samples may be assigned to the embedding of chitosan in HAp structure or to the interaction between CaP and Ch. These findings highlight the influence of the substrate temperature during the RF-MS deposition on the formation of HAp_CS composite coatings. We suppose that the deposition of chitosan by MAPLE on the CaP layers obtained by RF-MS technique at various temperatures of the substrates favors the formation of HAp_CS composite layers.

### 3.3. X-ray Diffraction

**Figure 13:**
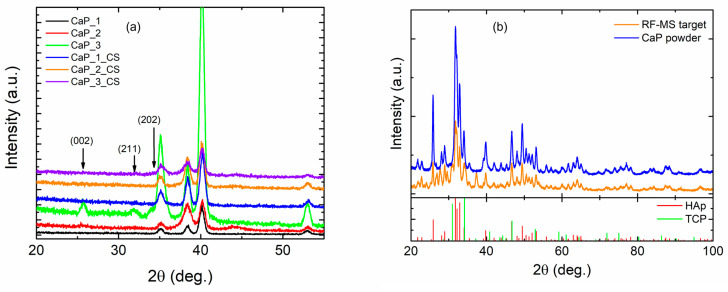
XRD results of (**a**) all samples deposited at different substrate temperatures and (**b**) initial CaP powder and sputtering target after preparation of samples.

From the GIXRD results presented in Figure 13a, it is observed that calcium phosphate layer crystallized only in the CaP_3 sample were the diffraction planes (002), (211), and (202) are highlighted as weak peaks along with the XRD peaks corresponding to Ti substrate. This observation agrees with the deposition parameters given that the CaP_3 sample was prepared at the highest substrate temperature. Furthermore, the post-deposition of chitosan breaks the crystalline structural order achieved during the synthetization of the CaP_3 layer. To show the structural stability of the sputtering target, we analyzed the calcium phosphate initial powder and pressed target after preparation of samples. By comparison of the two XRD patterns from Figure 13b, it is observed that sputtering target retains the initial structural phases of the CaP powder-HAp (ICDD 01-074-0565) and tricalcium diphosphate-TCP (ICDD 01-082-8142).

### 3.4. Scanning Electron Microscopy

#### Surface

**Figure 14:**
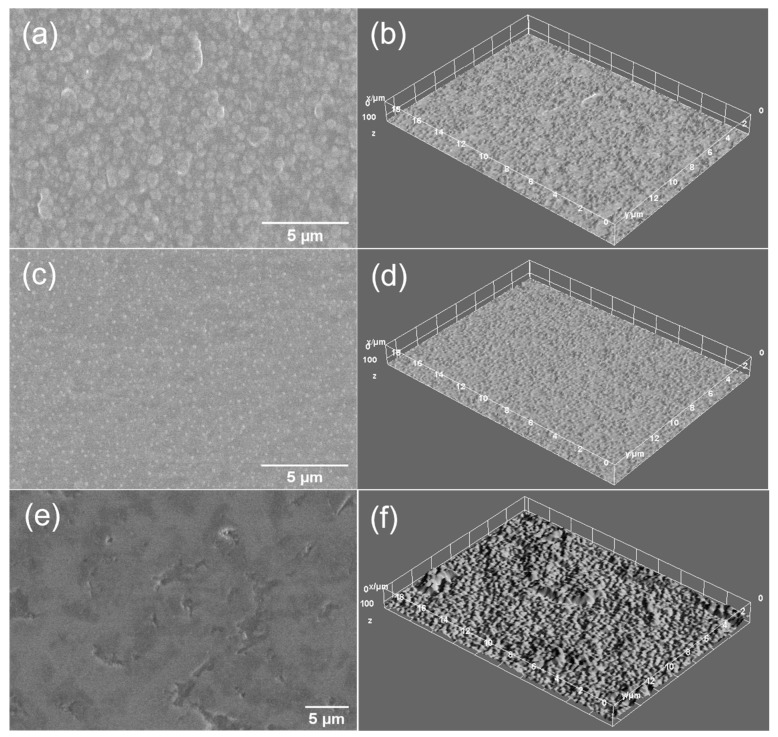
SEM images of (**a**) CaP_1, (**c**) CaP_2, (**e**) CaP_3 samples. 3D surface plots of (**b**) CaP_1, (**d**) CaP_2, (**f**) CaP_3 samples.

**Figure 15:**
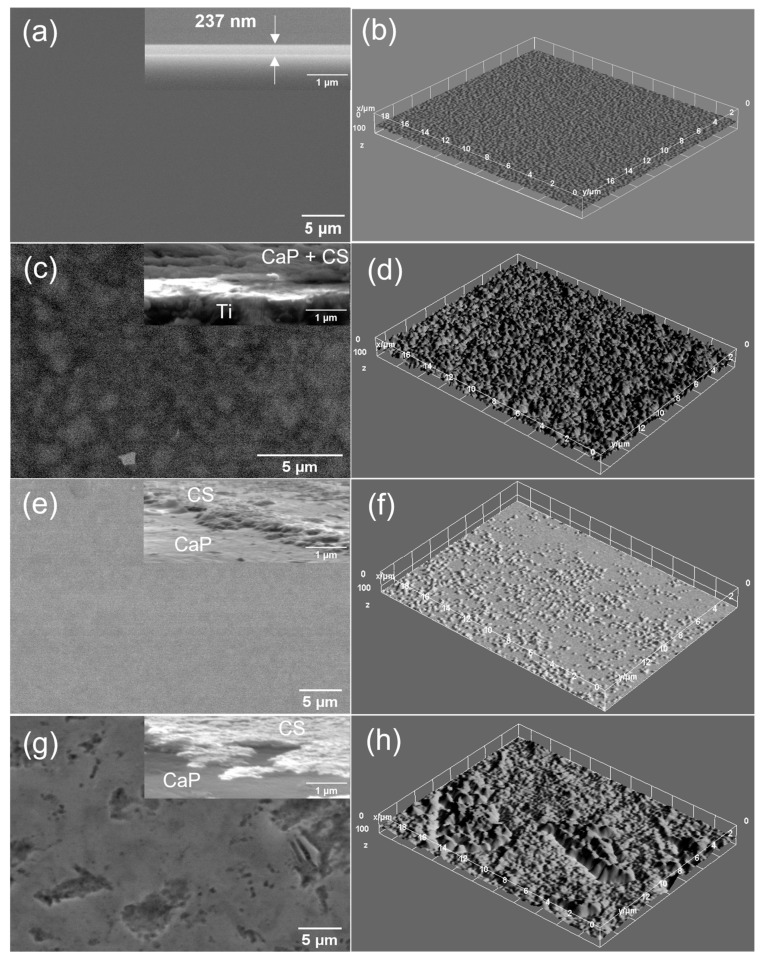
SEM images of (**a**) chitosan deposited on Si by MAPLE, (**c**) CaP_1_CS, (**e**) CaP_2_CS, and (**g**) CaP_3_CS samples. 3D surface plots of (**b**) chitosan deposited on Si by MAPLE, (**d**) CaP_1_CS, **(f**) CaP_2_CS, and (**h**) CaP_3_CS samples.

The SEM images of the CaP and CaP_CS layers are presented in Figure 14 and Figure 15, respectively. The surface morphology of the CaP coatings is dependent on the substrate temperature during the RF-MS deposition (Figure 14a,c,e).

A grain-like morphology was observed for the CaP_1 coating (Figure 14a). This morphology was previously reported for CaP films deposited by magnetron sputtering on Ti substrates [62]. No significant morphology changes were observed on the CaP_1_CS (Figure 15c). However, the SEM image indicates that chitosan entered between the grains, leading to a more compact surface. This is also sustained by the cross-section SEM image of the CaP_1_CS sample (Figure 15c), in which the CaP and the chitosan layers are combined.

As the substrate temperature was increased during the CaP deposition, smoother and more compact surfaces were obtained (Figure 14c,e). However, at 300 °C (CaP_3), micrometric cavities are formed. After chitosan deposition, the surface is smoother for CaP_2_CS (Figure 15e), while no differences were observed for CaP_3_CS. (Figure 15g). The cross-section SEM images of the CaP_2_CS (Figure 15e) and CaP_3_CS samples (Figure 15g) show that chitosan does not enter in the CaP layer, as the layers can be observed separately.

The surface textures of the CaP and CaP_CS layers can be better visualized in the 3D surface plots presented in Figure 14b,d,f and Figure 15b,d,f,h, respectively.

### 3.5. Energy Dispersive X-ray Spectroscopy

The elemental composition of the CaP and CaP_CS layers was determined by EDX.

#### 3.5.1. EDX Analysis of CaP Coatings

**Figure 16:**
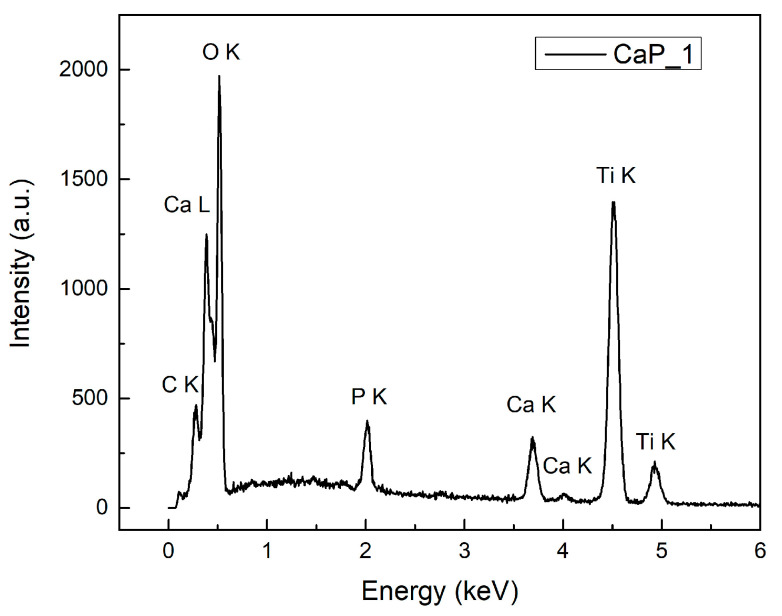
EDX spectrum of CaP_1.

Chemical elements specific for CaPs were identified for all CaP layers: Ca, P, and O. Small amounts of C, from the environment contamination, and Ti, specific for the substrate, were also observed in the EDX spectra. The EDX spectrum for the CaP_1 is presented in Figure 16.

The Ca/P ratios for the CaP layers are presented in Table 5. The Ca/P ratio increased form 1.69 for CaP_1 to 1.86 for CaP_2 and 1.93 for CaP_3, as the temperature of the substrate during the RF-MS deposition was increased. The Ca/P ratio for CaP_1 is close to the Ca/P ratio of stoichiometric HAp (Ca/P = 1.67). The Ca/P ratios for CaP_2 and CaP_3 are higher than 1.67. A similar trend was previously reported for the CaP layers deposited by RF-MS [15,63,64,65]. Nelea et al. associated the increase of Ca/P ratio with the presence of CaO in the films deposited by RF-MS. They mentioned that the presence of CaO increases the amount of Ca contained in the layers [66]. We suppose that oxidations occur probably due to the high temperature from the substrate during magnetron sputtering deposition process.

#### 3.5.2. EDX Analysis of CaP_CS Coatings

**Figure 17:**
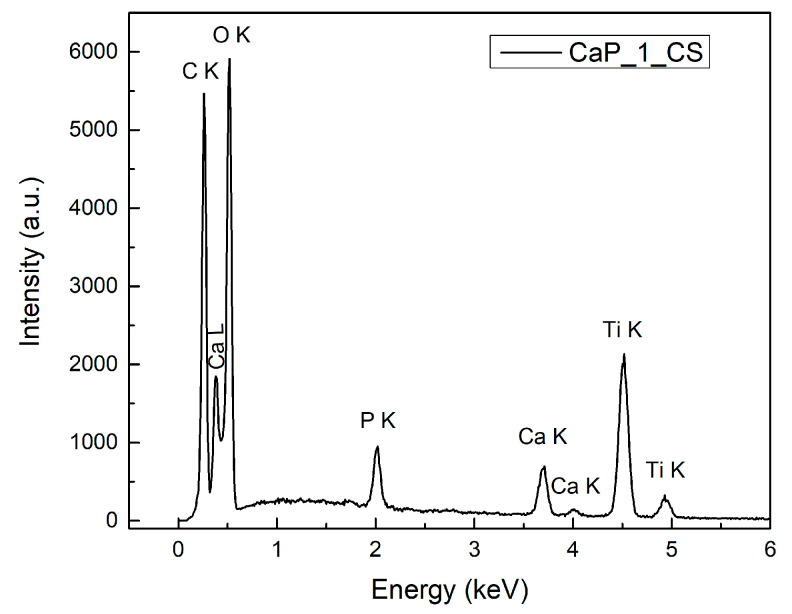
EDX spectrum of CaP_1_CS.

Chemical elements specific for CaPs (Ca, P, and O) and for chitosan (C and N) were identified in the EDX spectra of the CaP_CS layers, along with Ti. The EDX spectrum for the CaP_1_CS is presented in Figure 17.

The Ca/P ratios for the CaP_CS layers are presented in Table 6. The Ca/P ratio in-creased from 1.57 for CaP_1_CS to 1.70 and 1.97 for CaP_2_CS and CaP_3_CS, respectively. The trend of Ca/P ratio increasement with the temperature of the substrate during the RF-MS deposition process of CaP layers as that observed for the CaP layers is maintained. Additionally, the Ca/P ratio decreased from 1.69 (CaP_1) to 1.57 (CaP_1_CS) and from 1.86 (CaP_2) to 1.70 (CaP_2_CS). We can explain these values by considering the free radicals such as OH- and H+ formed during the evaporation of chitosan by MAPLE technique. When attaining the CaP layers, they can generate several physico-chemical processes inside them. We suppose that HPO_4_^2−^ may be formed in the CaP_1_CS composite layer and consecutively a calcium-deficient HAp is generated (Ca_10−x_(HPO_4_)_x_(PO_4_)_6−x_(OH)_2−x_, 0≤ x ≤ 1). In calcium-deficient HAp structures, the Ca/P ranges from 1.5 to 1.67 [3]. The fact that the FTIR analysis of CaP_1_CS coatings indicated the presence of HPO_4_^2−^ in the layer volume sustained our assumptions.

The Ca/P ratio of CaP_3_CS (1.97) slightly increases from 1.93 (CaP_3), possible due to CaO formation at CaP/CS interface. It may indicate that the chemical structure of the CaP layer was not affected during the chitosan deposition. This can be sustained by the SEM investigation (Figure 15e,g), which suggests that no major morphology changes appeared at the surface of the CaP layer deposited at a substrate temperature of 300 °C after the chitosan deposition. Moreover, the similar values of CaP_3 and CaP_3_CS suggest that the embedding of chitosan into CaP layer do not take place.

### 3.6. SEM and EDX Analysis of CaP and CaP_CS Layers Adhesion by Tape Tests

**Figure 18:**
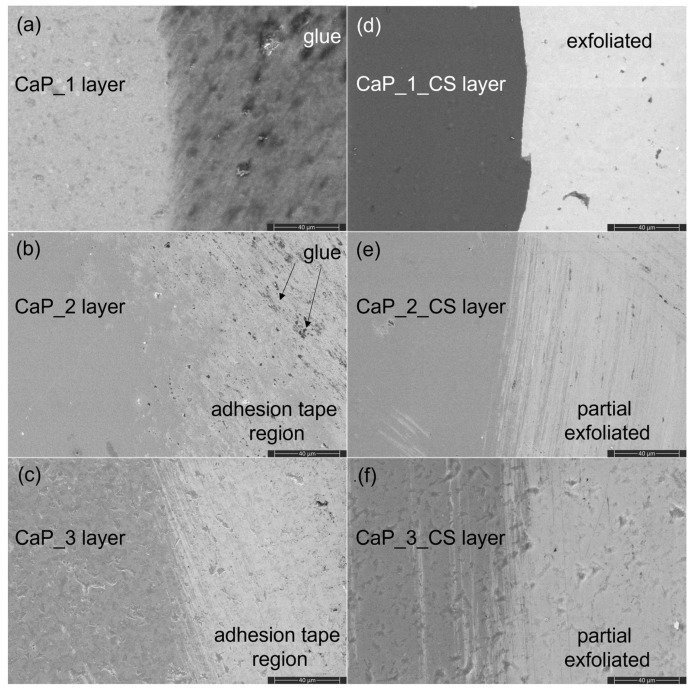
SEM images of (**a**) CaP_1, (**b**) CaP_2, (**c**) CaP_3, (**d**) CaP_1_CS, (**e**) CaP_2 _CS, and (**f**) CaP_3_CS samples after adhesion tests.

Figure 18 presents the surface morphology of the CaP and CaP_CS layers after the adhesion tests. The samples were also analyzed by EDX. The results showed that the CaP layers (Figure 18a–c) are not exfoliated from the substrate. For the CaP_1 layer, the glue from the adhesion tape remains attached on the surface (Figure 18a), while for the CaP_2 (Figure 18b) and CaP_3 (Figure 18c) the surface morphology of the layers is not modified. Therefore, we can ascertain the adherence between CaP and Ti.

The adhesion tests were also conducted on CaP_CS layers (Figure 18d–f). The EDX results suggest that the CaP_1_CS layer is partial exfoliated, as the percentages of C, O, Ca, and P decreased, while the Ti atomic percentage increased in the adhesion tape region (Figure 18d, right). For the CaP_2_CS (Figure 18e) and CaP_3_CS (Figure 18f) the EDX measured percentages suggest that only the chitosan from the CaP layer is partial exfoliated, as the percentage of C and N slightly decreased, while the percentage of O, P, and Ca were almost unchanged.

This is in agreement with the cross-section SEM images of the CaP_CS layers (Figure 15), which suggest that chitosan was completely embedded in the CaP_1 layer, while for the CaP_2_CS and CaP_3_CS, it remained partially at the CaP surface.

The CaP_CS composite layers are promising for implant (dental and orthopedic) coating applications. The bone is composed of an inorganic part (60%—mainly HAp), an organic part (30%), and water (10%) [67]. For bone tissue engineering, CaPs were intensively studied as promising materials for implant coatings. However, these materials cannot mimic the complex composition of bones. In this regard, composite materials based on CaPs and biopolymers were developed over the years in order to mimic the inorganic and the organic parts of the bone. Chitosan gained a lot of interest due to its antibacterial properties and biodegradability. Moreover, it improves the adhesion and the proliferation of osteoblasts, along with the matrix formation [60,67].

Visan et al. successfully obtained chitosan/biomimetic apatite coatings on Ti using the MAPLE technique. They tested the antimicrobial properties on two strains: Gram positive (*S. aureus*) and Gram negative (*E. coli*). The results showed that a more pronounced inhibitory effect on the biofilm development was observed for the Gram-positive strain [8]. Ansari et al. obtained chitosan/HAp nanocomposite coatings on Ti_6_Al_4_V alloy using the sol-gel process. They tested the bioactivity potential in an SBF solution in order to highlight that bone-like apatite can be formed at the surface of the coatings, which is essential for the implant-bone bonding. The results revealed that all samples (different HAp amounts) showed nucleation sites. They also tested cell proliferation using human mesenchymal stem cells and the results showed no cytotoxic effects of the chitosan/HAp coatings [59]. Zhang et al. obtained chitosan/HAp coatings, through an electrochemical deposition, on porous Ti_6_Al_4_V implants and conducted in vitro and in vivo studies. The alkaline phosphatase (ALP) activity assay results proved that the ALP activity is higher for the chitosan/HAp coated titanium than for uncoated titanium. The authors concluded that porous titanium coated with chitosan/HAp composite promote the proliferation and differentiation of osteoblast-like MC3T3-E1 cells (in vitro) and osteointegration (in vivo) [68]. Li et al. obtained HAp/chitosan coatings on titanium by micro-arc oxidation followed by the dip-coating method. They tested the biological and the antibacterial properties of these coatings, with promising results for implant applications. The SBF soaking test revealed that HAp coating promotes the formation of more nucleation sites when compared to the chitosan layers. Moreover, the cell proliferation was faster for the HAp coatings than for HAp/chitosan and the cell number decreased on the HAp/chitosan coatings as the chitosan concentration was increased. However, an increased amount of chitosan in the coatings determined enhanced antibacterial properties. Therefore, in order to improve both biological and antibacterial properties, HAp and chitosan amounts must be carefully adjusted [69]. Considering the results reported in the scientific literature regarding the efficiency of CaP_CS composite coatings for bone tissue engineering and despite the fact that the deposition techniques reported were different, we assume that the CaP_CS coatings deposited by combining the RF-MS and MAPLE techniques are promising for dental and orthopedic applications. 

## 4. Conclusions

Calcium phosphate–chitosan composite layers were obtained by combining two deposition techniques: radio-frequency magnetron sputtering and matrix-assisted pulsed laser evaporation. The substrate temperature during the deposition of CaP layers influences the morphological and chemical properties of the CaP and CaP_CS layers, subsequently.

The XPS investigation revealed that beside HAp, CaO and HPO_4_ were formed at the surface of the CaP layers either during the magnetron sputtering deposition process or after the layers were taken out from the vacuum deposition chamber. The results are in accordance with FTIR and EDX analysis. The FTIR investigation confirmed that HAp was successfully deposited on Ti substrates. The peak fitting analysis reveals that the substrate temperature increase induces chemical and physical modifications in the CaP layers. The EDX investigation revealed the increase of the Ca/P ratio with the substrate temperature, most probably due to the presence of CaO. The substrate temperature also influences the surface morphology of the CaP layers, from a grain-like morphology to a smoother and more compact surface, as the temperature increases. The layers have a high adherence strength to the Ti substrates.

For the CaP_CS layers, the XPS and FTIR investigations confirmed that chitosan was successfully deposited by MAPLE. A possible PO_4_^3−^/CO_3_^2−^ substitution was observed. The results indicated that chitosan was embedded in the CaP layer generated by RF-MS at ambient temperature. For the CaP layers obtained by heating the substrate, chitosan was deposited mainly at the surface. These results are confirmed by SEM images, which suggest that chitosan entered between the grains of CaP_1_CS, while no significant differences were observed for CaP_3_CS. The Ca/P ratio for CaP_1_CS and CaP_2_CS decreased when compared to the initial Ca/P ratio of CaP_1 and CaP_2, which could suggest several physico-chemical processes inside the CaP layers during the chitosan deposition by MAPLE. The deposition of chitosan on the CaP_3 layers destroys its crystallinity, as shown by GIXRD analysis. When compared to the CaP layers, the CaP_CS layers have a lower adherence to the substrate, with no complete exfoliation.

Further biological investigations such as osteoblasts adhesion, proliferation, and differentiation—real-time PCR, cell viability—MTT assay, microbial viability, and biofilm development will be performed in order to analyze the efficiency of the CaP and CaP_CS layers in bone tissue engineering applications. Moreover, the biological performance of our composite layers can be improved in the future by obtaining ion-substituted CaP layers and/or by adding growth factors and/or antibiotics during the MAPLE deposition of the chitosan layers.

## Figures and Tables

**Table 1:** XPS binding energies (eV) for the CaP coatings.

XPS Peaks	CaP_1 (eV)	CaP_2 (eV)	CaP_3 (eV)
Ca 2p_3/2_	347.2; 348.4	347; 348.3	347; 348.3
Ca 2p_1/2_	350.7; 351.9	350.5; 351.8	350.5; 351.8
P 2p_3/2_	133.1	132.9	132.9
P 2p_1/2_	134	133.75	133.8
O 1s	530.4	530.4	530.4
	531.2	531.1	531
	532.6	532.6	532.7
	534.1	534.3	534.6
C 1s	284.6	284.6	284.6
	286.3	286.3	286.3
	288.5	288.1	288.5
Ti 2p	461.9	461.9	461.9

**Table 2:** XPS binding energies (eV) for the CaP_CS coatings.

XPS Lines	CaP_1_CS (eV)	CaP_2_CS (eV)	CaP_3_CS (eV)
Ca 2p	350.7; 354.2	-	-
P 2p	135.3	-	-
O 1s	531.2	531.1	531
	532.3	532.3	532.1
	533.2	533.2	532.7
C 1s	284.6	284.6	284.6
	285.7	285.9	285.9
	286.8	286.7	286.9
	288.8	288.3	288.7
N 1s	399	399.1	399.1
	400	400	400.5
	402.5	402.9	404.5
Ti 2p	460.4; 466.2	-	-

**Table 5:** EDX Ca/P ratios for the CaP coatings.

**CaP_1**	**CaP_2**	**CaP_3**
1.69 ± 0.02	1.86 ± 0.04	1.93 ± 0.09

**Table 6:** EDX Ca/P ratios for the CaP_CS coatings.

**CaP_1_CS**	**CaP_2_CS**	**CaP_3_CS**
1.57 ± 0.02	1.70 ± 0.05	1.97± 0.12

## Data Availability

The data presented in this study are available on request from the corresponding author.

## References

[1] Habraken W., Habibovic P., Epple M., Bohner M. (2016). Calcium Phosphates in Biomedical Applications: Materials for the Future?. Mater. Today.

[2] Eliaz N., Metoki N. (2017). Calcium Phosphate Bioceramics: A Review of Their History, Structure, Properties, Coating Technologies and Biomedical Applications. Materials.

[3] Berzina-Cimdina L., Borodajenko N. (2012). Research of Calcium Phosphates Using Fourier Transform Infrared Spectroscopy. Infrared Spectroscopy—Materials Science, Engineering and Technology.

[4] Brånemark P.I., Breine U., Adell R., Hansson B.O., Lindström J., Ohlsson A. (1969). Intra-Osseous Anchorage of Dental Prostheses: I. Experimental Studies. Scand. J. Plast. Reconstr. Surg..

[5] Duraisamy R., Ganapathy D., Shanmugam R. (2021). Applications of Chitosan in Dental Implantology-a Literature Review. Int. J. Dent. Oral Sci..

[6] Wang J., Van Apeldoorn A., De Groot K. (2006). Electrolytic Deposition of Calcium Phosphate/Chitosan Coating on Titanium Alloy: Growth Kinetics and Influence of Current Density, Acetic Acid, and Chitosan. J. Biomed. Mater. Res. Part A.

[7] Aranaz I., Alcántara A.R., Civera M.C., Arias C., Elorza B., Caballero A.H., Acosta N. (2021). Chitosan: An Overview of Its Properties and Applications. Polymers.

[8] Visan A., Stan G.E., Ristoscu C., Popescu-Pelin G., Sopronyi M., Besleaga C., Luculescu C., Chifiriuc M.C., Hussien M.D., Marsan O. (2016). Combinatorial MAPLE Deposition of Antimicrobial Orthopedic Maps Fabricated from Chitosan and Biomimetic Apatite Powders. Int. J. Pharm..

[9] Xu Z., Neoh K.G., Lin C.C., Kishen A. (2011). Biomimetic Deposition of Calcium Phosphate Minerals on the Surface of Partially Demineralized Dentine Modified with Phosphorylated Chitosan. J. Biomed. Mater. Res. -Part B Appl. Biomater..

[10] Osman R.B., Swain M.V. (2015). A Critical Review of Dental Implant Materials with an Emphasis on Titanium versus Zirconia. Materials.

[11] Ma X., Gao Y., Zhao D., Zhang W., Zhao W., Wu M., Cui Y., Li Q., Zhang Z., Ma C. (2022). Titanium Implants and Local Drug Delivery Systems Become Mutual Promoters in Orthopedic Clinics. Nanomaterials.

[12] Groza A., Dreghici D.B., Ganciu M. (2019). Calcium Phosphate Layers Deposited on Thermal Sensitive Polymer Substrates in Radio Frequency Magnetron Plasma Discharge. Coatings.

[13] Goreninskii S.I., Bogomolova N.N., Malchikhina A.I., Golovkin A.S., Bolbasov E.N., Safronova T.V., Putlyaev V.I., Tverdokhlebov S.I. (2017). Biological Effect of the Surface Modification of the Fibrous Poly(L-Lactic Acid) Scaffolds by Radio Frequency Magnetron Sputtering of Different Calcium-Phosphate Targets. Bionanoscience.

[14] Surmenev R., Vladescu A., Surmeneva M., Ivanova A., Braic M., Grubova I., Cotrut C.M. (2017). Radio Frequency Magnetron Sputter Deposition as a Tool for Surface Modification of Medical Implants. Modern Technologies for Creating the Thin-film Systems and Coatings.

[15] Surmenev R.A., Surmeneva M.A., Grubova I.Y., Chernozem R.V., Krause B., Baumbach T., Loza K., Epple M. (2017). RF Magnetron Sputtering of a Hydroxyapatite Target: A Comparison Study on Polytetrafluorethylene and Titanium Substrates. Appl. Surf. Sci..

[16] Predoi D., Iconaru S.L., Predoi M.V., Groza A., Gaiaschi S., Rokosz K., Raaen S., Negrila C.C., Prodan A.M., Costescu A. (2020). Development of Cerium-Doped Hydroxyapatite Coatings with Antimicrobial Properties for Biomedical Applications. Coatings.

[17] Arnell R.D., Kelly P.J. (1999). Recent Advances in Magnetron Sputtering. Surf. Coat. Technol..

[18] Negut I., Grumezescu V., Grumezescu A.M., Bîrcă A.C., Holban A.M., Urzica I., Avramescu S.M., Gălăţeanu B., Hudiţă A. (2020). Nanostructured Thin Coatings Containing Anthriscus Sylvestris Extract with Dual Bioactivity. Molecules.

[19] Mihailescu N., Stan G.E., Duta L., Chifiriuc M.C., Bleotu C., Sopronyi M., Luculescu C., Oktar F.N., Mihailescu I.N. (2016). Structural, Compositional, Mechanical Characterization and Biological Assessment of Bovine-Derived Hydroxyapatite Coatings Reinforced with MgF2 or MgO for Implants Functionalization. Mater. Sci. Eng. C.

[20] Li X.Y., Nan K.H., Shi S., Chen H. (2012). Preparation and Characterization of Nano-Hydroxyapatite/Chitosan Cross-Linking Composite Membrane Intended for Tissue Engineering. Int. J. Biol. Macromol..

[21] Yang S., Zhang J. (2017). Matrix-Assisted Pulsed Laser Evaporation (MAPLE) Technique for Deposition of Hybrid Nanostructures. Front. Nanosci. Nanotechnol..

[22] Grumezescu V., Grumezescu A.M., Ficai A., Negut I., Vasile B.Ș., Gălățeanu B., Hudiță A. (2022). Composite Coatings for Osteoblast Growth Attachment Fabricated by Matrix-Assisted Pulsed Laser Evaporation. Polymers.

[23] Negut I., Ristoscu C., Tozar T., Dinu M., Parau A.C., Grumezescu V., Hapenciuc C., Popa M., Stan M.S., Marutescu L. (2022). Implant Surfaces Containing Bioglasses and Ciprofloxacin as Platforms for Bone Repair and Improved Resistance to Microbial Colonization. Pharmaceutics.

[24] Groza A., Surmeian A. (2015). Characterization of the Oxides Present in a Polydimethylsiloxane Layer Obtained by Polymerisation of Its Liquid Precursor in Corona Discharge. J. Nanomater..

[25] (2009). Standard Test Methods for Measuring Adhesion by Tape Test.

[26] López E.O., Mello A., Sendão H., Costa L.T., Rossi A.L., Ospina R.O., Borghi F.F., Silva Filho J.G., Rossi A.M. (2013). Growth of Crystalline Hydroxyapatite Thin Films at Room Temperature by Tuning the Energy of the RF-Magnetron Sputtering Plasma. ACS Appl. Mater. Interfaces.

[27] Major G.H., Fairley N., Sherwood P.M.A., Linford M.R., Terry J., Fernandez V., Artyushkova K. (2020). Practical Guide for Curve Fitting in X-Ray Photoelectron Spectroscopy. J. Vac. Sci. Technol. A.

[28] Boyd A.R., O’Kane C., Meenan B.J. (2013). Control of Calcium Phosphate Thin Film Stoichiometry Using Multi-Target Sputter Deposition. Surf. Coat. Technol..

[29] Uskoković V. (2020). X-Ray Photoelectron and Ion Scattering Spectroscopic Surface Analyses of Amorphous and Crystalline Calcium Phosphate Nanoparticles with Different Chemical Histories. Phys. Chem. Chem. Phys..

[30] Okawa S., Homma K., Kanatani M., Watanabe K. (2009). Characterization of Calcium Phosphate Deposited on Valve Metal by Anodic Oxidation with Polarity Inversion. Dent. Mater. J..

[31] Moulder J.F., Stickle W.F., Sobol P.E., Bomben K.D., Chastain J. (1992). Handbook of X-ray Photoelectron Spectrsocopy: A Reference Book of Standard Spectra for Identification and Interpretation of Xps Data.

[32] Praserthdam S., Rittiruam M., Maungthong K., Saelee T., Somdee S., Praserthdam P. (2020). Performance Controlled via Surface Oxygen-Vacancy in Ti-Based Oxide Catalyst during Methyl Oleate Epoxidation. Sci. Rep..

[33] Dreghici D.B., Butoi B., Predoi D., Iconaru S.L., Stoican O., Groza A. (2020). Chitosan–Hydroxyapatite Composite Layers Generated in Radio Frequency Magnetron Sputtering Discharge: From Plasma to Structural and Morphological Analysis of Layers. Polymers.

[34] Bensalem S., Hamdi B., Del Confetto S., Iguer-Ouada M., Chamayou A., Balard H., Calvet R. (2017). Characterization of Chitosan/Montmorillonite Bionanocomposites by Inverse Gas Chromatography. Colloids Surfaces A Physicochem. Eng. Asp..

[35] Saman N., Johari K., Kong H., Mohtar S.S., Hassan O., Ali N., Mat H. (2019). Enhanced Elemental Mercury Removal by Facile Sulfurization of Agrowaste Chars. Chem. Eng. Res. Des..

[36] Vandecandelaere N., Rey C., Drouet C. (2012). Biomimetic Apatite-Based Biomaterials: On the Critical Impact of Synthesis and Post-Synthesis Parameters. J. Mater. Sci. Mater. Med..

[37] Nguyen N.K., Leoni M., Maniglio D., Migliaresi C. (2013). Hydroxyapatite Nanorods: Soft-Template Synthesis, Characterization and Preliminary in Vitro Tests. J. Biomater. Appl..

[38] Delgado-López J.M., Iafisco M., Rodríguez I., Tampieri A., Prat M., Gómez-Morales J. (2012). Crystallization of Bioinspired Citrate-Functionalized Nanoapatite with Tailored Carbonate Content. Acta Biomater..

[39] Coates J. (2006). Interpretation of Infrared Spectra, A Practical Approach. Encyclopedia of Analytical Chemistry.

[40] Drabczyk A., Kudłacik-Kramarczyk S., Głab M., Kedzierska M., Jaromin A., Mierzwiński D., Tyliszczak B. (2020). Physicochemical Investigations of Chitosan-Based Hydrogels Containing Aloe Vera Designed for Biomedical Use. Materials.

[41] Queiroz M.F., Melo K.R.T., Sabry D.A., Sassaki G.L., Rocha H.A.O. (2015). Does the Use of Chitosan Contribute to Oxalate Kidney Stone Formation?. Mar. Drugs.

[42] Soares L.D.S., Perim R.B., de Alvarenga E.S., Guimarães L.D.M., Teixeira A.V.N.D.C., dos Reis Coimbra J.S., de Oliveira E.B. (2019). Insights on Physicochemical Aspects of Chitosan Dispersion in Aqueous Solutions of Acetic, Glycolic, Propionic or Lactic Acid. Int. J. Biol. Macromol..

[43] Radwan-Pragłowska J., Janus Ł., Piątkowski M., Sierakowska A., Matysek D. (2020). ZnO Nanorods Functionalized with Chitosan Hydrogels Crosslinked with Azelaic Acid for Transdermal Drug Delivery. Colloids Surf. B Biointerfaces.

[44] Li J. (2009). Structural Characterisation of Apatite-like Materials. Master’s Thesis.

[45] Albu P., Vlase G., Vlase T. (2015). Study of Bioactive Hydroxyapatite/Gelatin Composite. Part I - Synthesis and Characterization of the Material. New Front. Chem..

[46] Butler D.H., Shahack-Gross R. (2017). Formation of Biphasic Hydroxylapatite-Beta Magnesium Tricalcium Phosphate in Heat Treated Salmonid Vertebrae. Sci. Rep..

[47] Samantaray S.K., Parida K.M. (2003). Effect of Anions on the Textural and Catalytic Activity of Titania. J. Mater. Sci..

[48] Iconaru S.L., Predoi D., Ciobanu C.S., Motelica-Heino M., Guegan R., Bleotu C. (2022). Development of Silver Doped Hydroxyapatite Thin Films for Biomedical Applications. Coatings.

[49] Popa C.L., Ciobanu C.S., Voicu G., Vasile E., Chifiriuc M.C., Iconaru S.L., Predoi D. (2015). Influence of Thermal Treatment on the Antimicrobial Activity of Silver-Doped Biological Apatite. Nanoscale Res. Lett..

[50] Asaduzzaman S.M. (2017). Extraction of Hydroxyapatite from Bovine and Human Cortical Bone by Thermal Decomposition and Effect of Gamma Radiation: A Comparative Study. Int. J. Complement. Altern. Med..

[51] Stanislavov A.S., Sukhodub L.F., Sukhodub L.B., Kuznetsov V.N., Bychkov K.L., Kravchenko M.I. (2018). Structural Features of Hydroxyapatite and Carbonated Apatite Formed under the Influence of Ultrasound and Microwave Radiation and Their Effect on the Bioactivity of the Nanomaterials. Ultrason. Sonochem..

[52] Mróz W., Bombalska A., Budner B., Burdyńska S., Jedyński M., Prokopiuk A., Menaszek E., Ścisłowska-Czarnecka A., Niedzielska A., Niedzielski K. (2010). Comparative Study of Hydroxyapatite and Octacalcium Phosphate Coatings Deposited on Metallic Implants by PLD Method. Appl. Phys. A Mater. Sci. Process..

[53] Tsiourvas D., Tsetsekou A., Arkas M., Diplas S., Mastrogianni E. (2011). Covalent Attachment of a Bioactive Hyperbranched Polymeric Layer to Titanium Surface for the Biomimetic Growth of Calcium Phosphates. J. Mater. Sci. Mater. Med..

[54] Szatkowski T., Kołodziejczak-Radzimska A., Zdarta J., Szwarc-Rzepka K., Paukszta D., Wysokowski M., Ehrlich H., Jesionowski T. (2015). Synthesis and Characterization of Hydroxyapatite/Chitosan Composites. Physicochem. Probl. Miner. Process..

[55] Danilchenko S.N. (2009). Chitosan–Hydroxyapatite Composite Biomaterials Made by a One Step Co-Precipitation Method: Preparation, Characterization and in Vivo Tests. J. Biol. Phys. Chem..

[56] El-Sayed E.S.M., Omar A., Ibrahim M., Abdel-Fattah W.I. (2009). On the Structural Analysis and Electronic Properties of Chitosan/Hydroxyapatite Interaction. J. Comput. Theor. Nanosci..

[57] Ibrahim M., Abdel-Fattah W.I., El-Sayed E.-S.M., Omar A. (2013). A Novel Model for Chitosan/Hydroxyapatite Interaction. Quantum Matter.

[58] Xianmiao C., Yubao L., Yi Z., Li Z., Jidong L., Huanan W. (2009). Properties and in Vitro Biological Evaluation of Nano-Hydroxyapatite/Chitosan Membranes for Bone Guided Regeneration. Mater. Sci. Eng. C.

[59] Ansari Z., Kalantar M., Soriente A., Fasolino I., Kharaziha M., Ambrosio L., Raucci M.G. (2020). In-Situ Synthesis and Characterization of Chitosan/Hydroxyapatite Nanocomposite Coatings to Improve the Bioactive Properties of Ti6Al4V Substrates. Materials.

[60] Venkatesan J., Kim S.K. (2010). Chitosan Composites for Bone Tissue Engineering—An Overview. Mar. Drugs.

[61] Chatterjee S., Gupta A., Mohanta T., Mitra R., Samanta D., Mandal A.B., Majumder M., Rawat R., Singha N.R. (2018). Scalable Synthesis of Hide Substance-Chitosan-Hydroxyapatite: Novel Biocomposite from Industrial Wastes and Its Efficiency in Dye Removal. ACS Omega.

[62] Surmeneva M.A., Surmenev R.A., Nikonova Y.A., Selezneva I.I., Ivanova A.A., Putlyaev V.I., Prymak O., Epple M. (2014). Fabrication, Ultra-Structure Characterization and in Vitro Studies of RF Magnetron Sputter Deposited Nano-Hydroxyapatite Thin Films for Biomedical Applications. Appl. Surf. Sci..

[63] Pichugin V.F., Surmenev R.A., Shesterikov E.V., Ryabtseva M.A., Eshenko E.V., Tverdokhlebov S.I., Prymak O., Epple M. (2008). The Preparation of Calcium Phosphate Coatings on Titanium and Nickel–Titanium by Rf-Magnetron-Sputtered Deposition: Composition, Structure and Micromechanical Properties. Surf. Coat. Technol..

[64] Surmeneva M.A., Chaikina M.V., Zaikovskiy V.I., Pichugin V.F., Buck V., Prymak O., Epple M., Surmenev R.A. (2013). The Structure of an RF-Magnetron Sputter-Deposited Silicate-Containing Hydroxyapatite-Based Coating Investigated by High-Resolution Techniques. Surf. Coat. Technol..

[65] Surmenev R.A., Ryabtseva M.A., Shesterikov E.V., Pichugin V.F., Peitsch T., Epple M. (2010). The Release of Nickel from Nickel–Titanium (NiTi) Is Strongly Reduced by a Sub-Micrometer Thin Layer of Calcium Phosphate Deposited by Rf-Magnetron Sputtering. J. Mater. Sci. Mater. Med..

[66] Nelea V., Morosanu C., Iliescu M., Mihailescu I.N. (2004). Hydroxyapatite Thin Films Grown by Pulsed Laser Deposition and Radio-Frequency Magnetron Sputtering: Comparative Study. Appl. Surf. Sci..

[67] Fraile-martínez O., García-montero C., Coca A., Álvarez-mon M.A., Monserrat J., Gómez-lahoz A.M., Coca S., Álvarez-mon M., Acero J., Bujan J. (2021). Applications of Polymeric Composites in Bone Tissue Engineering and Jawbone Regeneration. Polymers.

[68] Zhang T., Zhang X., Mao M., Li J., Wei T., Sun H. (2020). Chitosan/Hydroxyapatite Composite Coatings on Porous Ti6Al4V Titanium Implants: In Vitro and in Vivo Studies. J. Periodontal Implant Sci..

[69] Li B., Xia X., Guo M., Jiang Y., Li Y., Zhang Z., Liu S., Li H., Liang C., Wang H. (2019). Biological and Antibacterial Properties of the Micro-Nanostructured Hydroxyapatite/Chitosan Coating on Titanium. Sci. Rep..

